# Epidemiologic and environmental characterization of the Re-emergence of St. Louis Encephalitis Virus in California, 2015–2020

**DOI:** 10.1371/journal.pntd.0010664

**Published:** 2022-08-08

**Authors:** Mary E. Danforth, Robert E. Snyder, Tina Feiszli, Teal Bullick, Sharon Messenger, Carl Hanson, Kerry Padgett, Lark L. Coffey, Christopher M. Barker, William K. Reisen, Vicki L. Kramer

**Affiliations:** 1 California Department of Public Health, Vector-Borne Disease Section, Richmond and Sacramento, California; 2 California Department of Public Health, Viral and Rickettsial Disease Laboratory, Richmond, California; 3 Department of Pathology, Microbiology and Immunology, School of Veterinary Medicine, University of California, Davis, California, United States of America; Florida Department of Health, UNITED STATES

## Abstract

St. Louis encephalitis virus (SLEV) is an endemic flavivirus in the western and southeastern United States, including California. From 1938 to 2003, the virus was detected annually in California, but after West Nile virus (WNV) arrived in 2003, SLEV was not detected again until it re-emerged in Riverside County in 2015. The re-emerging virus in California and other areas of the western US is SLEV genotype III, which previously had been detected only in Argentina, suggesting a South American origin. This study describes SLEV activity in California since its re-emergence in 2015 and compares it to WNV activity during the same period. From 2015 to 2020, SLEV was detected in 1,650 mosquito pools and 26 sentinel chickens, whereas WNV was detected concurrently in 18,108 mosquito pools and 1,542 sentinel chickens from the same samples. There were 24 reported human infections of SLEV in 10 California counties, including two fatalities (case fatality rate: 8%), compared to 2,469 reported human infections of WNV from 43 California counties, with 143 fatalities (case fatality rate: 6%). From 2015 through 2020, SLEV was detected in 17 (29%) of California’s 58 counties, while WNV was detected in 54 (93%). Although mosquitoes and sentinel chickens have been tested routinely for arboviruses in California for over fifty years, surveillance has not been uniform throughout the state. Of note, since 2005 there has been a steady decline in the use of sentinel chickens among vector control agencies, potentially contributing to gaps in SLEV surveillance. The incidence of SLEV disease in California may have been underestimated because human surveillance for SLEV relied on an environmental detection to trigger SLEV patient screening and mosquito surveillance effort is spatially variable. In addition, human diagnostic testing usually relies on changes in host antibodies and SLEV infection can be indistinguishable from infection with other flaviviruses such as WNV, which is more prevalent.

## Author summary

St. Louis encephalitis virus is a mosquito-borne virus that can cause human disease and is found in California, where it was detected every year from 1938 to 2003. However, after West Nile virus arrived in 2003, St. Louis encephalitis virus was not detected again until 2015, when it re-emerged in Riverside County. From 2015 through 2020, St. Louis encephalitis virus has been detected in mosquito pools and sentinel chicken sera samples in 16 counties and a total of 24 human disease cases have been reported. However, during that same time-period, West Nile virus has been detected in 10 times as many mosquito pools and 60 times as many chicken sera samples across 54 counties and over 2,400 human disease cases have been reported. Although mosquitoes are tested routinely for both viruses, surveillance is not uniform throughout the state, and there has been a steady decline in the use of sentinel chickens. Since St. Louis encephalitis virus patient screening is dependent upon environmental detection, California may be underestimating the incidence of human disease due to this virus.

## Introduction

St. Louis encephalitis virus (SLEV) is an arthropod-borne flavivirus that is maintained and amplified in an enzootic transmission cycle involving mosquitoes in the genus *Culex* and various bird species. Humans become infected with SLEV after being fed on by an infected female mosquito. The virus was first isolated from humans during an outbreak of SLEV disease in St. Louis County, Missouri in 1933 [1] and from *Culex* mosquitoes in Yakima Valley, Washington in 1939 [2].

The primary SLEV mosquito vectors in California are *Culex tarsalis*, *Cx*. *pipiens* and *Cx*. *quinquefasciatus* [3]. Common bird hosts for SLEV in California include house finches, house sparrows, and nestling mourning doves [4,5]. Similar to most arboviruses that cause central nervous system (CNS) disease in humans, most SLEV infections are asymptomatic or mild, with symptom onset 5–15 days after exposure [6] and exhibiting a broad range of clinical presentations [3]. SLEV infection has a 6% case fatality rate, most often in elderly or immunocompromised patients [6].

California’s mosquito-borne arbovirus surveillance program was initiated in response to epidemics of disease caused by SLEV and western equine encephalomyelitis virus in the state’s Central Valley in the mid-20^th^ century and is a collaboration among the California Department of Public Health (CDPH), the University of California, Davis Arbovirus Research and Training Laboratory (DART) [formerly the Center for Vectorborne Diseases, CVEC], as well as local vector control agencies (hereafter VCAs) and public health agencies throughout California. The current iteration of this program employs surveillance for arboviral infection in mosquitoes, sentinel chickens, dead wild birds, and disease in equids and humans. Adult chickens (>18 weeks old) are ideal SLEV sentinels; they do not develop viremias sufficient to infect mosquitoes but produce long-lasting SLEV neutralizing antibodies at readily detectable titers [7]. However, SLEV is not as virulent in wild birds as WNV [5] and is not pathogenic to horses [8].

In California, antibodies that neutralized SLEV were first identified in people with CNS disease in 1934 [9]. It is likely that SLEV disease previously had occurred in California, but these infections were undetected or conflated with infectious poliomyelitis. The first major recognized SLEV outbreak in the state occurred in 1937 when a reported 102 cases occurred in residents of 16 California counties—primarily in the state’s Central Valley [10]. From 1945 through 1969, 477 cases of SLEV were reported in California, predominantly in the Central Valley (a high of 97 among Kern County residents), dropping to a total of 97 from 1970 through 1989 [11], and only 10 between 1990 and 2003 [12]. Although human infections historically have been reported sporadically, enzootic SLEV activity has been documented annually in California from 1938 through 2003 [13]. Most environmental detections during 1970–2003 were from Imperial and Riverside counties, though Kern continued to report the most human cases during that time period [14], even though residents of southeastern California were frequently positive for antibodies [15].

After West Nile virus (WNV), another flavivirus in the Japanese encephalitis serocomplex, arrived in California in 2003 [16], the burden of WNV disease almost immediately surpassed the cumulative 584 SLEV clinical disease cases that had been reported since 1945, with 791 WNV infections reported in 2004 alone. SLEV subsequently disappeared completely from California, despite substantial increases in the amount and frequency of environmental surveillance, that included testing for SLEV RNA in pooled mosquitoes [17]. From 1993 through 2002, less than 5,000 mosquito pools were tested annually. In 2003, approximately 10,000 mosquito pools were tested and from 2005 through 2014, more than 20,000 pools were tested annually, increasing to more than 40,000 by 2020 [18]. The most likely explanation for the disappearance of SLEV, based on laboratory studies, is that WNV may have provided cross-protection against SLEV infection in common sylvatic avian hosts, thus inhibiting SLEV’s transmission and causing its local extinction in California [16, 19].

In 2015, SLEV re-emerged in Riverside County’s Coachella Valley, with multiple detections in both mosquitoes and sentinel chickens [20]. These detections coincided with an outbreak of 19 human cases of SLEV disease in Arizona [21]. Genetic analyses showed that the closest archived sequences to the re-emerged strain of SLEV were from Argentina and although the virus was undetected until the 2015 outbreak, it likely was introduced in 2013 and was found in an archived mosquito pool collected in Maricopa County, Arizona, in 2014 [22]. Subsequently, from 2016 through 2020, 16 more counties in southern and central California also detected SLEV activity via mosquitoes, sentinel chickens, and/or reported human cases.

SLEV is now considered a re-emerging threat in California; however, human surveillance has been hampered by clinical symptoms that are indistinguishable from WNV, a poor understanding of who should be tested for SLEV, a high rate of asymptomatic infections, and limited arboviral diagnostic tools. Herein, we describe SLEV surveillance data in California since 2015 and contextualize both the limitations of human surveillance for SLEV as well as the need for more uniform collection of environmental data to support directed interventions that reduce the risk of human arboviral disease.

## Methods

### Ethics statement

Title 17, Section 2500 of the California Code of Regulations specifies which data must accompany WNV and SLEV case reports to the California Department of Public Health (CDPH). These data are stored in a secure location where access is restricted to authorized CDPH staff. Analysis of human surveillance data is routine public health surveillance and exempt from Institutional Review Board review and approval. Enzootic surveillance data are the property of the agencies that generate them. These data were obtained through a VectorSurv data request #000047 submitted on 1/7/2021 to the California Vectorborne Disease Surveillance System [23].

### SLEV Surveillance in california

Title 17, Section 2500 of the California Code of Regulations mandates reporting of human SLEV- and WNV-positive diagnostic test results to the local health department responsible for the jurisdiction where the patient resides. These health departments then conduct investigations and report cases to CDPH that fulfill the Council of State and Territorial Epidemiologists’ (CSTE) arboviral disease case definition [24]. Hospitalization, co-morbidity, and other patient metadata were abstracted from those reports.

Enzootic arboviral surveillance in California has been described in detail elsewhere [18, 25–27]. Most mosquito pools in California include up to 50 mosquitoes and are tested concurrently for WNV, SLEV, and western equine encephalitis virus using a triplex RT-PCR assay [27]. SLEV is detected using a set of SLEV-specific TaqMan assay primers and probes:

primer 1—SLE2420: F, 5’-CTGGCTGTCGGAGGGATTCT -3’;

primer 2—SLE2487c: F, 5’- TAGGTCAATTGCACATCCCG– 3’;

SLE2444-probe: F, 5’- TCTGGCGACCAGCGTGCAAGCCG– 3’ [17, 28]. Seroconversion in sentinel chickens is detected using an enzyme immunoassay (EIA), with positives confirmed by comparative endpoint plaque reduction neutralization test (PRNT) or other serological assays [29, 30].

Since the re-emergence of SLEV in California in 2015, human surveillance for SLEV disease has been triggered by an environmental detection of SLEV in a county within a particular year. At that time, alerts were sent to healthcare providers to consider SLEV as a differential diagnosis when evaluating acute febrile or neuroinvasive infections of unknown origin. Local health departments then were encouraged to obtain specimens from individuals suspected to be infected with WNV or SLEV and submit them for additional testing at the CDPH Viral and Rickettsial Disease Laboratory (VRDL) (2016-present) or the United States Centers for Disease Control and Prevention (2016 and 2017), particularly for the first identified cases of WNV and/or SLEV in each county each year and all fatal cases of either virus. CDPH VRDL used either an SLEV immunofluorescent assay (IFA) or CDC-Developed IgM antibody capture enzyme-linked immunosorbent assay (MAC-ELISA) [31] to identify specific anti-SLEV IgM antibodies. Comparative endpoint PRNT on serum, cerebrospinal fluid (CSF), and blood samples is required for confirmation of SLEV infections in humans in California. These tests are available upon request to all California local health partners. However, there has been cross-reactivity between SLEV and WNV in all these diagnostic tests. When that occurs, the patient is diagnosed with whichever virus elicits the highest titer, or if the titers are the same, whichever virus is predominating in their county of residence. Blood collection agencies and organ procurement organizations do not screen for SLEV infections in California.

The California Vectorborne Disease Surveillance (VectorSurv) Gateway serves as a repository for enzootic arboviral surveillance data and provides web-based tools to local vector control agencies for real-time data management, reporting, visualization, and analysis [23, 32]. VectorSurv data are stored in a back-end PostgreSQL database with PostGIS for advanced data retrieval and aggregation [33].

### Analyses

SAS 14.3 (SAS Institute, Cary, NC, USA) was used for descriptive analyses, figures, and tables. The map was generated in ArcGIS Pro 2.8.0 (ESRI, Redlands, CA, USA). We described SLEV prevalence by sex, age, and county of residence. Annual disease incidence was estimated per 100,000 persons using population estimates from the California Department of Finance in 2021 [34]. Estimated disease onset dates were used to describe SLEV disease in California, whereas the dates of mosquito pool collections and sentinel chicken bleeds were used for enzootic data. The minimum infection rate (MIR) for WNV or SLEV within mosquito pools was calculated as the number of positive pools divided by the total number of mosquitoes tested and multiplied by 1,000.

## Results

### Environmental Surveillance for SLEV

The first detections of SLEV in California since 2003 were in four *Cx*. *tarsalis* mosquito pools collected on July 28, 2015 in Riverside County’s Coachella Valley. Following these initial detections, an additional 34 *Cx*. *tarsalis* pools and nine sentinel chickens from two flocks tested positive for SLEV infection. All positive samples from 2015 were collected near the Salton Sea in the southern Coachella Valley, an area with frequent historical SLEV detections, including in 2003 [13, 15, 35]. SLEV-positive mosquito pools were collected between July 28 and October 6, 2015, and sentinel chicken seroconversions to SLEV were detected between August 17 and November 9, 2015.

**Fig 1:**
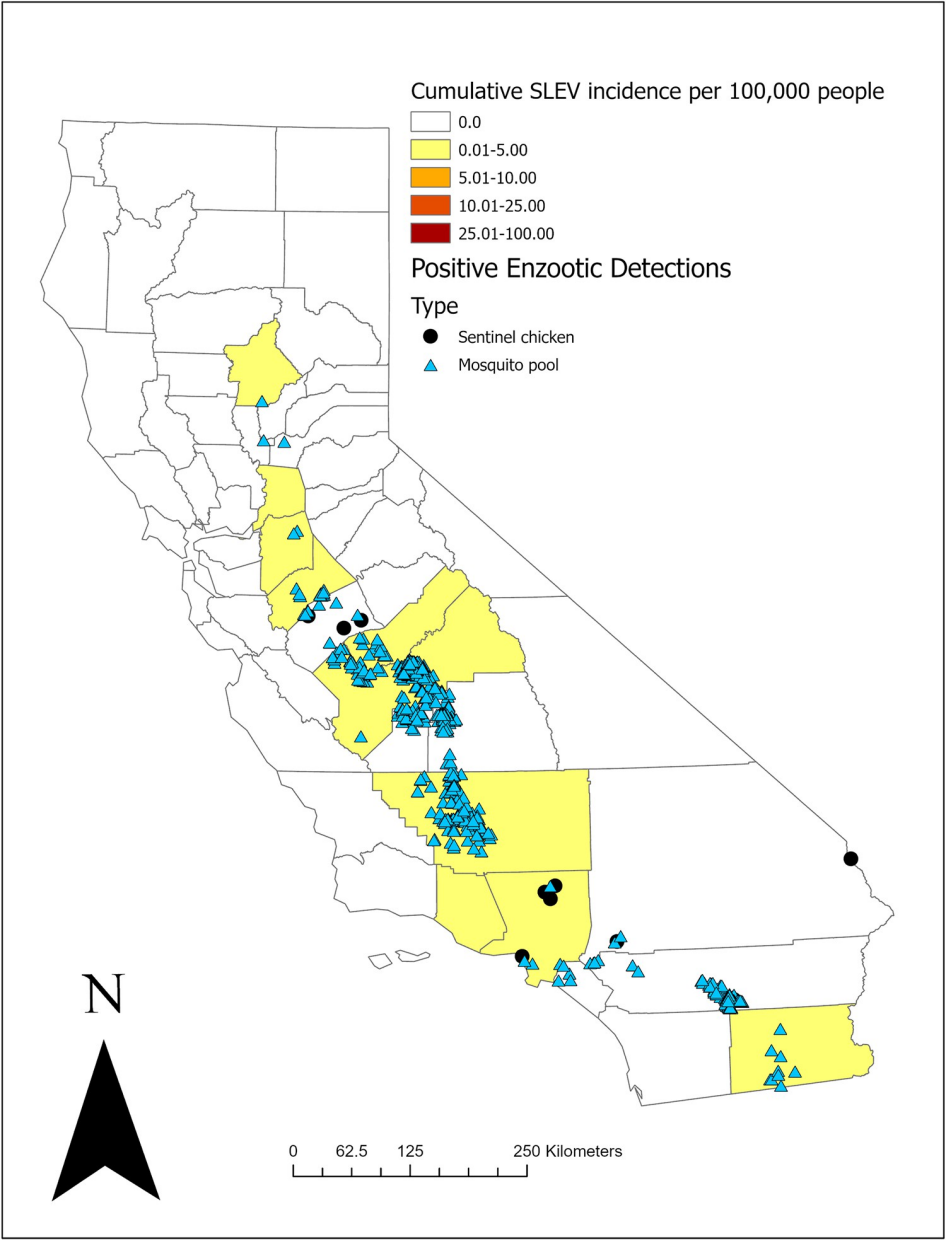
St. Louis encephalitis virus (SLEV) detections in California, 2015–2020. County data courtesy of U.S. Census Bureau TIGER/line spatial files, 2016. (Shapefile: https://www.census.gov/geographies/mapping-files/time-series/geo/tiger-line-file.2016.html License information: https://www2.census.gov/geo/pdfs/maps-data/data/tiger/tgrshp2021/TGRSHP2021_TechDoc_Ch1.pdf).

From 2015 to 2020, a total of 244,919 mosquito pools from 40 counties were tested for SLEV, with 1,650 positive pools reported from 16 (40%) counties (Fig 1, Table 1). The virus was detected in five *Culex* species: *Cx*. *quinquefasciatus*, *Cx*. *pipiens*, and their hybrids (922 pools, 56%), *Cx*. *tarsalis* (714 pools, 43%), *Cx*. *stigmatosoma* (13 pools, 1%), and *Cx*. *erythrothorax* (1 pool, <1%). Positive pools were detected from May 11 through November 13, with the greatest number (590, 36%) testing positive in September. The statewide annual MIR ranged from a low of 0.1 per 1,000 (2015) to a high of 0.4 (2020), with individual counties observing SLEV MIRs as high as 5.9 (2020, Imperial County).

**Table 1:** Mosquito pool detections of St. Louis encephalitis virus (SLEV) and West Nile virus (WNV) in California counties where SLEV was detected, 2015–2020. Number of positive *Culex spp*. pools (Mosquito Infection Rate per 1,000 tested).

County	2015		2016		2017		2018		2019		2020		Total	
	SLEV	WNV	SLEV	WNV	SLEV	WNV	SLEV	WNV	SLEV	WNV	SLEV	WNV	SLEV	WNV
Riverside	38 (0.3)	158 (1.1)	92 (0.5)	32 (0.2)	23 (0.1)	196 (1.1)	56 (0.3)	36 (0.2)	108 (0.4)	524 (2.1)	159 (0.8)	64 (0.3)	476 (0.4)	1,010 (0.9)
Fresno	0 (0)	108 (2.8)	1 (<0.1)	183 (5.0)	63 (1.6)	167 (4.2)	56 (1.1)	119 (2.4)	58 (0.9)	495 (7.9)	233 (2.7)	322 (3.7)	411 (1.3)	1,394 (4.5)
Kern	0 (0)	135 (6.1)	75 (3.0)	80 (3.2)	18 (0.6)	152 (5.1)	65 (2.1)	48 (1.5)	56 (1.5)	129 (3.6)	31 (1.0)	83 (2.7)	245 (1.4)	627 (3.6)
Kings	0 (0)	144 (7.3)	4 (0.3)	118 (7.3)	21 (1.4)	79 (5.3)	30 (3.5)	22 (2.5)	4 (0.3)	63 (3.8)	11 (0.6)	87 (4.4)	70 (0.9)	513 (5.3)
Los Angeles	0 (0)	294 (3.5)	2 (<0.1)	437 (4.1)	1 (<0.1)	582 (5.1)	1 (<0.1)	75 (0.6)	2 (<0.1)	93 (0.6)	0 (0)	437 (3.1)	6 (<0.1)	1918 (2.7)
Madera	0 (0)	21 (2.4)	3 (0.2)	103 (6.8)	10 (0.6)	62 (4.0)	14 (0.9)	55 (3.3)	5 (0.4)	85 (6.3)	17 (1.2)	77 (5.3)	49 (0.6)	403 (4.8)
Orange	NT	576 (3.6)	2 (<0.1)	444 (3.2)	0 (0)	280 (1.9)	0 (0)	96 (0.8)	3 (<0.1)	208 (1.6)	0 (0)	326 (2.2)	5 (<0.1)	1,930 (2.3)
San Bernardino	0 (0)	291 (5.6)	0 (0)	82 (0.9)	2 (<0.1)	295 (4.3)	0 (0)	12 (0.3)	4 (0.1)	51 (0.7)	0 (0)	12 (0.2)	6 (<0.1)	743 (1.9)
Tulare	0 (0)	528 (9.2)	1 (0.8)	260 (2.9)	6 (0.7)	630 (6.9)	162 (1.3)	77 (0.7)	96 (0.8)	813 (7.0)	45 (0.4)	189 (1.8)	310 (0.9)	2,497 (4.3)
Butte	0 (0)	94 (5.4)	0 (0)	48 (2.6)	1 (0.1)	47 (2.3)	0 (0)	48 (2.5)	0 (0)	44 (2.3)	0 (0)	28 (1.3)	1 (<0.1)	309 (2.6)
Imperial	NT	NT	0 (0)	0 (0)	3 (1.4)	0 (0)	3 (1.9)	1 (0.7)	5 (5.3)	2 (2.1)	10 (5.9)	3 (1.8)	21 (3.2)	6 (0.9)
Merced	0 (0)	7 (0.9)	0 (0)	12 (1.4)	2 (0.2)	40 (4.3)	0 (0)	12 (1.0)	2 (0.1)	48 (2.4)	0 (0)	42 (2.1)	4 (0.1)	161 (2.1)
Placer	NT	52 (2.1)	NT	103 (2.8)	1 (<0.1)	59 (1.6)	0 (0)	230 (5.4)	0 (0)	53 (1.1)	0 (0)	58 (1.6)	1 (<0.1)	555 (2.5)
Stanislaus	0 (0)	85 (1.0)	0 (0)	259 (4.7)	27 (0.49)	196 (3.6)	0 (0)	111 (1.5)	13 (0.2)	208 (3.3)	2 (<0.1)	351 (4.3)	42 (0.1)	1,210 (2.9)
Yuba	0 (0)	23 (4.7)	0 (0)	18 (3.7)	1 (0.2)	18 (2.7)	0 (0)	8 (1.4)	0 (0)	22 (3.4)	0 (0)	2 (0.3)	1 (<0.1)	91 (2.6)
San Joaquin	NT	208 (2.7)	NT	350 (5.2)	0 (0)	242 (1.95)	0 (0)	533 (4.4)	0 (0)	288 (3.4)	2 (<0.1)	260 (3.1)	2 (<0.1)	1,881 (3.4)
Sacramento	0 (0)	164 (2.3)	0 (0)	455 (4.9)	0 (0)	153 (1.6)	0 (0)	300 (3.2)	0 (0)	74 (1.2)	0 (0)	115 (1.7)	0 (0.0)	1,261 (2.6)
**California**	**38 (<0.1)**	**3,327 (3.2)**	**180 (0.2)**	**3,525 (3.0)**	**179 (0.2)**	**3,365 (2.7)**	**387 (0.3)**	**1,963 (1.6)**	**356 (0.26)**	**3,286 (2.4)**	**510 (0.4)**	**2,628 (2.0)**	**1,650 (0.2)**	**18,094 (2.4)**

In that same time-period, a total of 276,933 mosquito pools from 40 counties were tested for WNV, with 18,108 positive pools reported from 34 (85%) counties (Fig 1, Table 1). Typically, the same mosquito pools were tested for both SLEV and WNV, so the numbers tested generally represent the same mosquitoes, although a few local agencies tested only for WNV in certain years, making the total numbers tested lower for SLEV. WNV was detected from seven *Culex* species: *Cx*. *quinquefasciatus* (9,119 pools, 50.4%), *Cx*. *tarsalis* (6,077 pools, 33.6%), *Cx*. *pipiens* (2,690 pools, 14.9%), *Cx*. *stigmatosoma* (158 pools, <1%), *Cx*. *erythrothorax* (43 pools, <1%), *Cx*. *thriambus* (6 pools, <1%), and *Cx*. *restuans* (1 pool, <1%); three *Aedes* species: *Ae*. *aegypti* (6 pools, <1%), *Ae*. *albopictus* (1 pool, <1%), and *Ae*. *vexans* (2 pools, <1%); and two *Culiseta* species: *Cs*. *incidens* (4 pools, <1%), and *Cs*. *inornata* (1 pool, <1%). Positive pools were detected from January 29 through December 14, with the greatest number (6,772, 37%) testing positive in August. The statewide annual MIR in *Culex* species mosquitoes ranged from a low of 1.6 (2018) to a high of 3.2 (2015).

From 2015 to 2020, 58,648 sentinel chicken sera samples from 34 counties were screened for SLEV and WNV by EIA. Twenty-six (<1%) sentinel chickens from 4 (11%) counties tested positive for SLEV antibodies and 1,542 (3%) sentinel chickens from 26 (76%) counties tested positive for WNV antibodies (Fig 1, Table 2). Sentinel chicken seroconversions for SLEV occurred as early as July 12 (2016, San Bernardino County) and as late as November 9 (2015, Riverside County). For WNV, sentinel chicken seroconversions occurred as early as May 17 (2016, San Diego County) and as late as November 18 (2015, Los Angeles County). The greatest number of seroconversions for both viruses was detected in August (SLEV: 11, 42%; WNV: 630, 41%), The most seroconversions detected for SLEV were in 2015 and 2017, with 9 each year, while WNV peaked in 2015 with 448 seroconversions. There were multiple observations of counties and years where SLEV sentinel chicken seroconversions were detected without positive mosquito pools, and vice versa.

**Table 2:** Number of sentinel chicken seroconversions for St. Louis encephalitis virus (SLEV) and West Nile virus (WNV) in California counties where SLEV was detected, 2015–2020. NT = no flocks deployed or tested.

County	2015		2016		2017		2018		2019		2020		Total	
	SLEV	WNV	SLEV	WNV	SLEV	WNV	SLEV	WNV	SLEV	WNV	SLEV	WNV	SLEV	WNV
Riverside	9	22	NT	NT	NT	NT	NT	NT	NT	NT	NT	NT	9	22
Fresno	NT	NT	NT	NT	NT	NT	NT	NT	NT	NT	NT	NT	NT	NT
Kern	0	0	0	0	NT	NT	NT	NT	NT	NT	NT	NT	0	0
Kings	NT	NT	NT	NT	NT	NT	NT	NT	NT	NT	NT	NT	NT	NT
Los Angeles	0	137	2	126	2	145	0	30	0	28	0	38	4	504
Madera	NT	NT	NT	NT	NT	NT	NT	NT	NT	NT	NT	NT	NT	NT
Orange	NT	NT	NT	NT	NT	NT	NT	NT	NT	NT	NT	NT	NT	NT
San Bernardino	0	36	2	23	6	36	NT	NT	NT	NT	NT	NT	8	95
Tulare	NT	NT	NT	NT	NT	NT	NT	NT	0	10	0	10	0	20
Butte	0	37	0	38	0	31	0	37	0	34	0	23	0	200
Imperial	NT	NT	NT	NT	NT	NT	NT	NT	NT	NT	NT	NT	NT	NT
Merced	0	23	0	35	1	19	1	16	3	16	0	14	5	123
Placer	0	8	0	7	0	5	0	4	0	8	NT	NT	0	32
Stanislaus	0	9	NT	NT	NT	NT	NT	NT	NT	NT	NT	NT	0	9
Yuba	0	11	0	13	0	8	0	2	0	5	0	1	0	40
San Joaquin	NT	NT	NT	NT	NT	NT	NT	NT	NT	NT	NT	NT	NT	NT
Sacramento	0	2	0	3	0	2	0	5	0	4	0	4	0	20
**California**	**9**	**448**	**4**	**343**	**9**	**305**	**1**	**163**	**3**	**139**	**0**	**144**	**26**	**1,542**

### SLEV infections in humans

From 2015 to 2020, CDPH VRDL conducted 3,942 tests for SLEV via either MAC-ELISA, IFA, or PRNT from 1,150 individuals (S1 Table); 1,145 of the individuals were tested for both SLEV and WNV, whereas 5 were screened for only SLEV. Of these 3,942 tests, 2,974 (75%) were comparative endpoint PRNT for SLEV and WNV. There were 139 people tested via PRNT using both serum and CSF. There were 1,525 samples submitted from the 1,145 individuals tested for both viruses; 425 (32%) of 1320 samples were positive for both viruses by PRNT, along with 25 (12%) of 205 samples tested by MAC-ELISA or IFA. For those individuals who tested positive for both WNV and SLEV, they were classified as a case of whichever virus caused the higher titer or, when titers were equal, which virus predominated in the vicinity of the case’s residence. During this time period, VRDL testing identified 22 SLEV cases and CDC identified 2 additional cases. In addition to the 5 patients screened for SLEV only and the 1145 screened for both WNV and SLEV at VRDL, 284 were screened only for WNV. Not all WNV case patients were tested at VRDL; from 2015 to 2020, just 844 (34%) of the reported 2,469 WNV cases were tested by VRDL. Prior to the re-emergence of SLEV, from 2003 to 2014, 1,053 of 8,392 (13%) patients who had samples submitted for WNV also were screened for SLEV at VRDL and all were negative for SLEV

There were no human SLEV infections reported to CDPH in 2015, when the only environmental evidence of SLEV was detected in the southern Coachella Valley near the Salton Sea. During 2016–2020, 24 cases of SLEV disease were reported from 10 counties, with the most (7 cases, 29%; 0.68 infections/100,000 residents) reported from Fresno County (Fig 1, Table 3). The 5-year cumulative incidence of SLEV disease in California was 0.06/100,000 persons. There were 3–6 infections reported each year, with the earliest symptom onset occurring on July 1, and the latest on October 28. Eight (35%) infections had symptom onset in July. The median age of SLEV case-patients was 65.5 years (mean 64.1, range 31–90). Sixteen (67%) SLEV case-patients were male. Eighteen (75%) case-patients were white, 1 (4%) reported as “other,” with the remainder failing to report a race (n = 5, 21%). Four (17%) case-patients reported Hispanic ethnicity. Twenty-one (88%) case-patients presented with neuroinvasive symptoms during their illness, all of whom were hospitalized (Table 4). Two (9%) reported SLEV infections were fatal; both had neuroinvasive symptoms and were more than 65 years old. Among the 16 hospitalized patients with data available for length of stay (16/21, 76%), the median duration of hospitalization was 8 days (mean 10.4, range 4–26). Symptoms included encephalitis, meningitis, sepsis, and rhabdomyolysis. Sixteen (67%) case-patients had a comorbidity, including, but not limited to hypertension, diabetes, obesity, or cancer. Six (25%) case-patients with comorbidities were explicitly immunocompromised, with either a recent history of cancer, chronic bacterial infection, or a previously identified autoimmune disorder. Twenty-one cases were reported from counties that had concurrent environmental detections of SLEV; however, for only three of those cases the SLEV MIR was higher than the WNV MIR (Kern County-2018, Imperial County-2019 x2). There was one case of SLEV from Stanislaus County that occurred in a year without SLEV environmental detections (2018), but there were environmental detections in the preceding and following years. Two cases were reported in residents of counties that had no environmental detections of SLEV after the introduction of WNV: Sacramento County (2016) and Ventura County (2017). The case from Ventura County reported travel during the incubation period to California counties where SLEV was detected that year and is not considered to be exposed in Ventura. However, the Sacramento County case did not report travel during their incubation period to any location with known SLEV transmission.

**Table 3:** Number of human St. Louis encephalitis virus (SLEV) cases and cumulative incidence of SLEV disease cases by year, California, 2016–2020.–Indicates no detections.

County in California	2016	2017	2018	2019	2020	Total Cases	Cumulative Incidence (cases per 100K persons)
Butte	--	1	--	--	--	**1**	0.49
Fresno	1	--	1	2	3	**7**	0.68
Imperial	--	--	--	2	--	**2**	1.08
Kern	1	1	1	1	--	**4**	0.44
Los Angeles	--	--	2	--	--	**2**	0.02
Madera	--	--	--	--	1	**1**	0.63
Sacramento	1	--	--	--	--	**1**	0.06
San Joaquin	--	--	--	--	1	**1**	0.13
Stanislaus	--	1	1	1	1	**4**	0.72
Ventura	--	1	--	--	--	**1**	0.12
**Total (CA)**	**3**	**4**	**5**	**6**	**6**	**24**	**0.06**

**Table 4:** Clinical presentations of the 24 St. Louis encephalitis virus (SLEV) cases reported in California, 2016–2020.

	N (%)
Clinical presentation	
Neuroinvasive	21 (88%)
Non-neuroinvasive	3 (12%)
Hospitalized*	21 (88%)
Symptoms	
Fever	20 (83%)
Headache	16 (67%)
Myalgia	14 (58%)
Vomiting	11 (46%)
Stiff Neck	11 (46%)
Meningitis	9 (38%)
Sepsis	5 (21%)
Encephalitis	5 (21%)
Diarrhea	4 (17%)
Rhabdomyolysis	2 (10%)

* All hospitalized infections were neuroinvasive

**Fig 2:**
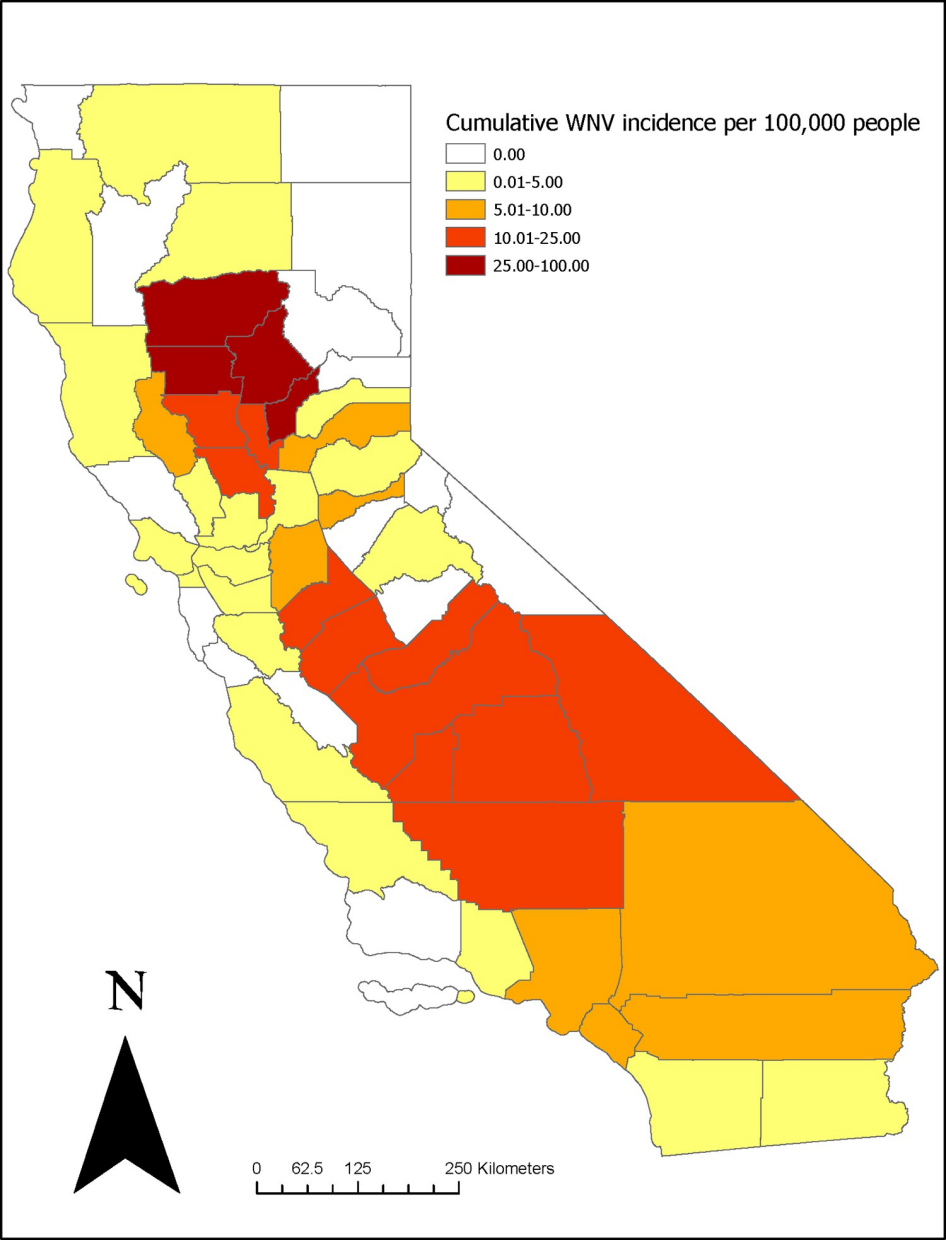
Human West Nile virus (WNV) cumulative incidence in California by county, 2015–2020. County data courtesy of U.S. Census Bureau TIGER/line spatial files, 2016. (Shapefile: https://www.census.gov/geographies/mapping-files/time-series/geo/tiger-line-file.2016.html. License information: https://www2.census.gov/geo/pdfs/maps-data/data/tiger/tgrshp2021/TGRSHP2021_TechDoc_Ch1.pdf).

During that same time-period, 2,469 symptomatic cases of WNV disease were reported from 43 counties (Fig 2), with a 5-year cumulative incidence for the state of 6.26/100,000 persons from 2016 to 2020. Each year 214–800 cases were reported, with peak onset during the months of August (487 cases, 20%) and September (457 cases, 19%). The median age of WNV case-patients was 60 years (mean 58.1, range 1–98) and 1,542 (62%) were male. Under the category of race, 1472 (60%) were reported as white and under ethnicity, and 599 (4%) were reported as Hispanic. Overall, 1,809 cases (73%) were classified as neuroinvasive, 2,028 cases (82%) were hospitalized, and 143 (6%) cases were fatal.

## Discussion

From 2015 to 2020, enzootic or human SLEV activity was reported in 17 (29%) of California’s 58 counties. Of these, only 10 counties (59%) identified and reported human cases of SLEV disease, most of which were severe neuroinvasive disease and/or in patients with underlying medical conditions. Although SLEV was detected in mosquito pools from 16 counties, the majority (1,085, 66%) were collected from the southern Central Valley (Fresno, Kern, Kings, Madera, and Tulare counties) and Riverside County (476, 29%). Less than five percent of enzootic SLEV activity was detected outside of these areas.

Because SLEV and WNV are transmitted by the same mosquito species, the timing and geographical distribution of SLEV disease cases mirrored WNV disease cases in California. The higher incidence of neuroinvasive disease among all SLEV disease cases may have been due to the increased risk in individuals with underlying conditions–which were reported in half of the SLEV disease patients. However, data describing underlying conditions among WNV case-patients in California were not collected consistently, so comparisons between these two diseases should be made with caution. To improve data collection and enable explicit comparisons of the clinical presentations of both diseases, we implemented a specific electronic case report form for SLEV in 2019 and developed infrastructure to aggregate clinical data from WNV case-patients. Nonetheless, the true magnitudes of WNV and SLEV infections remain poorly defined, and the likelihood of testing and diagnosis are influenced by such factors as social determinants of health [36, 37], availability of clinical specimens, the effectiveness of diagnostic tests, and, more recently, COVID-19 [38, 39].

Laboratory confirmation of clinical diagnoses and differentiation of SLEV from WNV is difficult. Among reported SLEV disease cases in California, 58% of patients had cross-reactive flavivirus neutralizing antibodies to WNV. Molecular assays that rely on detection of viral RNA show limited use for diagnosing arboviral disease because most patients are no longer viremic when they present clinical symptoms. Although paired convalescent/acute samples, and/or paired CSF and serum samples can facilitate differentiation of WNV from SLEV, CSF is usually not available for patients with non-neuroinvasive symptoms, and patients rarely return several weeks later for the collection of convalescent samples. Due to the cross-reactivity with WNV, there likely has been misclassification of some SLEV disease cases as WNV disease and vice versa; 30% of samples screened for WNV and SLEV tested positive for antibodies to both viruses. In addition, 66% of WNV disease cases reported to CDPH from 2015 through 2020 were tested only for WNV via EIA or IFA by commercial laboratories, without a sample available for PRNT at VRDL, which means that testing was inadequate to determine whether these cases might have been caused by SLEV infections. After the detection of SLEV within a particular county and year via mosquitoes or sentinel chickens, CDPH encourages local public health agencies to submit human samples to VRDL for SLEV testing, but specimen acquisition depends on local capacity to obtain them from commercial laboratories with limited specimen retention policies.

The first reported case of SLEV disease in California in 2016 was a resident of Sacramento County. Although robust arbovirus surveillance has been conducted in Sacramento County for decades, including consistent monitoring of mosquitoes and sentinel chickens for SLEV, SLEV has not been detected in mosquito pools or sentinel chicken sera since 1986 and the case patient did not report travel to an area where SLEV was endemic. It is possible this individual’s positive serology result was due to an anamnestic immune response following a WNV infection, although the patient had no record of a previous flavivirus infection [40, 41]. In house finches, experimental sequential infections with WNV following an SLEV infection markedly boosted antibodies to SLEV but not WNV [19].

Even though it is difficult to differentiate SLEV infections from WNV, treatment for people infected with either flavivirus is palliative and not influenced by a specific diagnosis. Similarly, because SLEV and WNV are transmitted by the same *Culex* mosquito species, prevention and control efforts targeting WNV are also effective at preventing SLEV transmission. For this reason, counties are encouraged to report suspect flavivirus infections to vector control partners as soon as possible rather than waiting for definitive differential PRNT results. Human infections can be reclassified and attributed to SLEV or WNV when adequate diagnostic testing results are available.

In contrast to WNV, for which the entire Central Valley and Southern California are at elevated risk of infection [18], the vast majority of enzootic SLEV activity was reported from Riverside County, largely from the Coachella Valley, and the southern Central Valley. The southern Central Valley has detected SLEV in mosquito pools every year since 2016, and 66% (1,085/1,650) of all positive pools were collected from this region. However, more human WNV disease than SLEV disease has been reported since 2016, even in years when the MIR in vector mosquito species was greater for SLEV than WNV [34, 42–46]. The genotype detected in the state since 2015 is different than those detected prior to 2003, which were usually limited to the southeastern deserts including Coachella Valley [47], with limited dispersal into the Southern Central Valley [14].

SLEV activity may have gone undetected in counties that do not perform sentinel chicken surveillance. Sentinel chickens may be more sensitive in detecting *Culex*-borne arboviral activity than mosquito pool testing because chickens are exposed to mosquitoes 24 hours per day, 7 days a week whereas traps collecting mosquitoes for testing are usually set overnight at weekly or biweekly intervals. However, due to the costs for upkeep and maintenance of chicken flocks, and delays in the timing of detecting seroconversions, there has been a steady decline in the use of chickens among VCAs, particularly in the regions of California with the greatest enzootic SLEV activity. From 1993 through 2002, an average of 180 flocks were deployed statewide each year; in 2005 the number of flocks had increased to a maximum of 262 in 40 counties, but by 2020 only 95 flocks were deployed across 23 counties, with few to no flocks in counties that had SLEV activity prior to 2003 [42–46]. Riverside County deployed no sentinel flocks after 2015 and only one flock was monitored in the southern Central Valley during 2019 and 2020 [42–46]. In those areas with chickens, the co-circulation of WNV also could hamper the detection of SLEV seroconversions due to cross-reactivity of WNV and SLEV neutralizing antibodies. Once a chicken has seroconverted to WNV or SLEV, it is typically not replaced, and would no longer be a useful sentinel for the other virus. These factors could result in the underreporting of SLEV activity, especially in areas without robust and geographically representative mosquito sampling.

Despite these limitations, the overall intensity of arboviral environmental surveillance in California substantially increased after the introduction of WNV and the incorporation of RT-PCR testing of mosquitoes beginning in 2003. However, that alone is not sufficient to explain why more enzootic SLEV activity is detected now than in the decades prior to SLEV’s disappearance in 2003. Studies on pre-2003 strains showed limited susceptibility and relatively low viremia levels in California birds [5] compared to WNV [48]. Changes in the structure and quantity of surveillance have inhibited direct comparisons of detections between these eras. Previously circulating SLEV strains did not kill experimentally infected wild birds [5], and there has not been a detection of SLEV in dead wild birds tested since 2015, despite routine screening as part of the WNV surveillance program. However, there have been no published studies comparing the virulence of the new SLEV strain to that of WNV in California or other southwestern US states, or to the strains of SLEV that circulated prior to the arrival of WNV.

The causes for the apparent extinction and then re-emergence of SLEV in California remain undetermined. Possibly the widespread infection of passerines and resulting cross-protective herd immunity following the 2003 invasion by WNV markedly reduced SLEV transmission, which was at a comparatively low level at that time [16]. It is also possible that sustained cross-immunity to SLEV in wild bird populations resulting from WNV infections could have made most SLEV strains less transmissible, thereby limiting the potential for re-establishment to strains that were less susceptible to cross-immunity, although this hypothesis has not been evaluated. The emergence of WNV in California was followed by increased funding for vector control [49] which likely led to more effective suppression of *Culex* vector populations, potentially contributing to the continued absence of SLEV. Concurrently, driven by persistent drought and limited water allotments, cropping strategies in the Central Valley have changed from flood-irrigated row crops to vineyards and orchards which produced less surface water [50] and may have decreased vector mosquito habitat. In addition, beginning in 2013, a new mosquito species of public health concern, *Aedes aegypti*, was detected in multiple locations in California [51]; this likely diverted mosquito control resources from *Culex* and WNV, and indirectly SLEV, to *Ae*. *aegypti* control. Collectively, some or all of these events may have enabled the re-emergence and persistence of SLEV. Ongoing surveillance and virus sequencing will continue to describe the temporal and evolutionary dynamics of these two flaviviruses with similar ecologies.

The extinction and reintroduction of SLEV in California is not unprecedented. Though SLEV was known to overwinter in the Salton Sea area [52, 53] and persisted in the Central Valley for at least 25 concurrent years, genomic tracing from different sites in California prior to 2003 indicated periodic extinction and reintroduction of the virus into the state [3, 54], similar to the apparent pattern since 2015, although SLEV’s apparent absences were shorter in prior decades. Since the re-emergence of SLEV in 2015, phylogeographic analyses indicated that repeated introductions of SLEV from Arizona into Southern California, without year-to-year persistence of the same viral strains [55]. Within Central California, continual SLEV activity since 2016 was likely the result of a single SLEV introduction that has persisted [55]. On a larger scale, there were at least two introductions of SLEV into the southwestern US, including California, before local spread [22].

## Conclusions

SLEV re-emerged in California in 2015 after an absence spanning more than a decade. Since 2016, there have been 24 reported cases of SLEV disease and environmental activity detected in 18 of California’s counties, with most activity in the southern Central Valley and Riverside County’s Coachella Valley. Differential diagnoses of human disease caused by SLEV and closely related WNV have been complicated by non-specific and cross-reactive diagnostic tests and small sample sizes, which likely contributed to underreporting of SLEV disease compared to more common WNV disease in California and perhaps other parts of the United States.

More research is needed to understand ecological interactions between SLEV and WNV. Given cross-immunity between these viruses and their overlap in key vectors and vertebrate hosts, each virus is likely to cause selection pressures that shape the viral population of the other. The long absence of SLEV from California from 2004 to 2014 and the continued co-existence of SLEV and WNV since 2015 indicate that the re-emergent SLEV strain may have competitive advantages that differ from those strains of prior decades that re-established more frequently.

Both WNV and SLEV can cause serious morbidity and mortality in humans and prevention for both is predicated on avoiding mosquito bites, such as using repellents and protective clothing. California residents and visitors, particularly in the Central Valley and Southern California, are encouraged to remain vigilant to the threat of mosquito-borne diseases, particularly in summer and early fall.

## Supporting information

S1 Table Number of patients tested for St. Louis encephalitis virus (SLEV) in serum, cerebrospinal fluid (CSF), whole blood, and other specimens, at the California Department of Public Health, by year, 2015–2020. (DOCX)
