# Supplementary material for: Epidemiologic and environmental characterization of the Re-emergence of St. Louis Encephalitis Virus in California, 2015–2020
Source: PLoS Negl Trop Dis. 2022 Aug 8;16(8):e0010664. doi: 10.1371/journal.pntd.0010664 (PMC9387929; doi:10.1371/journal.pntd.0010664)
Supplement: S1 Table — (DOCX) [file pntd.0010664.s001.docx]

**Supplemental Information Table 1:** Number of specimens from St. Louis encephalitis virus (SLEV) suspect patients tested for SLEV, at the California Department of Public Health, by year, 2015-2020

| **Test by Year** | **Specimen Type** | | | |
| --- | --- | --- | --- | --- |
|  | **Serum** | **CSF** | **Whole Blood** | **Total** |
| **2015** | **100** | **86** | **0** | **186** |
| EIA SLEV IgM | 98 | 85 | -- | 183 |
| PRNT WNV vs SLEV | 2 | 1 | -- | 3 |
| **2016** | **318** | **91** | **5** | **414** |
| EIA SLEV IgM | 2 | 2 | -- | 4 |
| PRNT WNV vs SLEV | 316 | 89 | 5 | 410 |
| **2017** | **898** | **148** | **3** | **1049** |
| EIA SLEV IgM | 219 | 24 | -- | 243 |
| PRNT WNV vs SLEV | 679 | 124 | 3 | 806 |
| **2018** | **618** | **137** | **0** | **755** |
| EIA SLEV IgM | 146 | 27 | -- | 173 |
| PRNT WNV vs SLEV | 472 | 110 | -- | 582 |
| **2019** | **736** | **315** | **0** | **1051** |
| EIA SLEV IgM | 138 | 48 | -- | 186 |
| IFA SLEV IgM | 65 | 33 | -- | 98 |
| PRNT WNV vs SLEV | 533 | 234 | -- | 767 |
| **2020** | **352** | **125** | **0** | **477** |
| EIA SLEV IgM | 48 | 35 | -- | 83 |
| PRNT WNV vs SLEV | 304 | 90 | -- | 394 |
| **2021** | **8** | **0** | **0** | **8** |
| EIA SLEV IgM | 2 | -- | -- | 2 |
| PRNT WNV vs SLEV | 6 | -- | -- | 6 |
| **Grand Total** | **3032** | **902** | **8** | **3942** |
